# The neural representation of Arabic digits in visual cortex

**DOI:** 10.3389/fnhum.2015.00517

**Published:** 2015-09-24

**Authors:** Lien Peters, Bert De Smedt, Hans P. Op de Beeck

**Affiliations:** ^1^Parenting and Special Education Research Unit, KU LeuvenLeuven, Belgium, Europe; ^2^Laboratory of Biological Psychology, KU LeuvenLeuven, Belgium, Europe

**Keywords:** fMRI, MVPA, visual cortex, Arabic digits, ventral visual stream

## Abstract

In this study, we investigated how Arabic digits are represented in the visual cortex, and how their representation changes throughout the ventral visual processing stream, compared to the representation of letters. We probed these questions with two functional magnetic resonance imaging (fMRI) experiments. In Experiment 1, we explored whether we could find brain regions that were more activated for digits than for number words in a subtraction task. One such region was detected in lateral occipital cortex. However, the activity in this region might have been confounded by string length—number words contain more characters than digits. We therefore conducted a second experiment in which string length was systematically controlled. Experiment 2 revealed that the findings of the first experiment were task dependent (as it was only observed in a task in which numerosity was relevant) or stimulus dependent (as it was only observed when the number of characters of a stimulus was not controlled). We further explored the characteristics of the activation patterns for digit and letter strings across the ventral visual processing stream through multi-voxel pattern analyses. We found an alteration in representations throughout the ventral processing stream from clustering based on amount of visual information in primary visual cortex (V1) towards clustering based on symbolic stimulus category higher in the visual hierarchy. The present findings converge to the conclusion that in the ventral visual system, as far as can be detected with fMRI, the distinction between Arabic digits and letter strings is represented in terms of distributed patterns rather than separate regions.

## Introduction

The vast majority of research on how numbers are processed in the brain has focused on the semantic representation of numbers, i.e., the magnitude a number represents. However, little is known about the visual processing of numbers, even though this type of processing has been claimed to be important when doing arithmetic (Dehaene and Cohen, 1995, 1997; Menon, 2015). We focused on the ventral visual processing stream, because this pathway plays a role in the identification and categorization of visual objects, such as digits. We investigated which regions in the visual cortex were activated whilst participants calculated with numbers in symbolic (e.g., Arabic digits and number words) and non-symbolic (dot arrays) formats.

The most commonly used theoretical framework to study number processing and arithmetic is the Triple Code Model (Dehaene and Cohen, 1995, 1997). According to this model, three distinct codes of numerical information can be activated. First, the *visual* code is involved in processing Arabic number forms, and in recognizing and discriminating number-letter strings. This process is assumed to take place in the inferior ventral occipito-temporal areas. Second, there is an analogue quantity or *magnitude* code which represents, estimates and compares numerosities (Ansari, 2008; Nieder and Dehaene, 2009), and is implicated when we manipulate numerosities, as during arithmetic (Simon et al., 2002; Dehaene et al., 2003; Piazza et al., 2007; Eger et al., 2009; Bulthé et al., 2014). This code is located in the inferior parietal areas, and more specifically in the intraparietal sulci. Third, there is a *verbal* code in which numbers are represented by words. This code is located in the left-hemispheric perisylvian areas and in the left angular gyrus (Dehaene et al., 2003) and is implicated in symbolic number processing (Price and Ansari, 2011) and in accessing memory of arithmetic facts (Delazer et al., 2003; Grabner et al., 2009). A recent meta-analysis by Arsalidou and Taylor (2011) confirmed these regions as being involved in arithmetic, but they suggested to update this model by including regions such as frontal areas, cerebellum, insula and cingulate cortex.

Although the roles of the magnitude and verbal codes have been extensively studied, the *visual* code has not been studied much in the context of arithmetic (Menon, 2015). In their description of the Triple Code Model, Dehaene and Cohen (1995, 1997) suggested that this code should be located in occipito-temporal regions, along the visual ventral processing stream, which plays a role in identification and categorization of objects (Mishkin et al., 1983; Goodale and Milner, 1992). There is a large body of studies that directly investigated the coding of objects (Grill-Spector et al., 2001; Reddy and Kanwisher, 2006), faces (Halgren et al., 1999; Rossion et al., 2003), and even words (Cohen and Dehaene, 2004; Baker et al., 2007) in this ventral visual pathway (see also Malach et al., 2002; Grill-Spector and Malach, 2004). However, similar data on the coding of numerical symbols, such as Arabic digits, have not been reported. Dehaene and Cohen (1995) stated that, similar to a visual region specifically tuned for letter strings (visual word form area, VWFA), there should also be region specifically tuned for digits, hosting the visual code. Furthermore, studies have shown that occipito-temporal regions are activated together with the IPS during arithmetic (Rickard et al., 2000; Zago et al., 2001; Keller and Menon, 2009; Wu et al., 2009; Rosenberg-Lee et al., 2011), invigorating Dehaene and Cohen’s claim that also visual regions in the occipito-temporal areas are involved in arithmetic. This hypothesis is further backed by results from a recent meta-analysis of studies using number processing or arithmetic tasks in healthy adult samples. Specifically, Arsalidou and Taylor (2011) found that the left fusiform gyrus is involved in number processing and arithmetic tasks, and that the left inferior occipital gyrus is implicated in subtraction tasks.

Only a small number of studies specifically attempted to find a brain region that could host this visual number processing code, a so-called *visual number form area* (Dehaene and Cohen, 1995; Menon, 2015), yet their findings are mixed. For example, Park et al. (2012) found a cluster of voxels in the right lateral occipital area that was activated more by number strings than by letter strings in the context of a visual matching task, during which participants were presented with either two letter strings or two digit strings, and had to decide whether the two strings were visually identical. Polk et al. (2002) conducted a study with a similar paradigm, in which participants passively viewed strings of consonants, strings of digits, strings of shapes and fixation points. They did not find a region that was more activated by digits than by letters. However, in a subset of individuals, they found various regions in the visual cortex, especially around the left fusiform gyrus and left inferior regions, which were more active when viewing digits than fixation points. Pinel and Dehaene (2013) observed a region in the right fusiform gyrus that was activated more when participants performed a number comparison task (i.e., deciding whether a number was larger or smaller than 65) with digits compared to with number words. Finally, using intracranial electrophysiological recordings, Shum et al. (2013) found a region in the inferior temporal gyrus that responded more to digits compared to morphologically, phonologically and semantically similar symbols.

The studies described above all focus on the role of the occipito-temporal cortex in number processing. However, although this brain area is also implicated in arithmetic (see above), previous studies with arithmetic tasks merely reported activity in the occipito-temporal cortex, but crucially, they did not look into the visual cortex specifically in the context of arithmetic (Rickard et al., 2000; Zago et al., 2001; Keller and Menon, 2009; Wu et al., 2009; Pinel et al., 2001) and thus did not clarify the role the visual cortex plays in arithmetic.

The current study serves two aims. First of all, we will investigate to what extent there is a focal region in the ventral visual system specifically tuned for Arabic digits that might host the visual code. Unlike previous studies, we will use an arithmetic paradigm with different formats to present numerosities for two reasons. First, activation in regions in the occipito-temporal cortex, where the visual code is thought to be located, has been found to be associated with arithmetic (see above). Second, including multiple formats (i.e., dot arrays, Arabic digits and number words) will allow us to isolate the visual code better by contrasting arithmetic with Arabic digits from arithmetic with number words. Both conditions include a symbolic and semantic format, and they mainly differ in the degree to which they are supposed to activate the visual code in the Triple Code Model.

Second of all, we will study the emergence and formation of this visual code by looking at the evolution of how digits are represented throughout the ventral visual processing stream. In order to do so, we will delineate three key regions along this ventral visual processing stream: primary visual cortex (V1), lateral occipital complex (LOC) and VWFA. We selected these regions because of their specific characteristics: V1 is the first visual processing region, LOC is specifically tuned to visual objects (Grill-Spector et al., 2001), and finally, VWFA responds highly to visually presented letter strings (Baker et al., 2007). Using multi-voxel pattern analysis, we will compare activation patterns of all conditions to investigate the similarity with which conditions are represented in those regions of interest, and how these similarities change throughout the ventral visual processing stream. A great advantage of multivariate analyses is that they can reveal differences between conditions that are possibly averaged out in univariate analyses (Norman et al., 2006; Raizada and Kriegeskorte, 2010).

## Material and Methods

### Participants

Twelve healthy Dutch-speaking university students and employees took part in this study (four males, aged between 18 and 38 years old, *M* = 24.7, all right-handed), which consisted of two functional magnetic resonance imaging (fMRI) experiments. All participants had normal or correct-to-normal vision, and reported no history of neurological or psychiatric illness. Participants gave written consent prior to taking part in the study, and were paid for their participation. The study was approved by the Medical Ethical Committee of KU Leuven.

### Apparatus

Imaging data were collected via a 3T Philips Intera Scanner, located at the Department of Radiology of the University Hospital in Leuven, with a 12-channel head coil. Functional images were collected with an EPI sequence (47 slices, 2 × 2 mm in plane voxel size, slice thickness 2 mm, interslice gap 1 mm, TR = 3000 ms, TE = 30 ms, flip angle = 90 degrees, 104 × 104 matrix). We acquired a high-resolution T1-weighted anatomical image (182 slices, resolution 0.98 × 0.98 × 1.2 mm, TR = 9.6 ms, TE = 4.6 ms, 256 × 256 acquisition matrix) for each participant. Stimuli were presented with PsychToolbox 3 (Brainard, 1997) and displayed via a Barco 6400i LCD projector onto a screen located approximately 35 cm from participants’ eyes, which was visible via a mirror attached to the head coil. Participants answered by pressing one of two response buttons on a response box, which they controlled with their right hand.

### Experimental Tasks

#### Experiment 1

In the first experiment, participants performed a subtraction task in which they were asked to subtract two magnitudes (up to 20), and to decide whether the result was larger or smaller than a reference magnitude. We manipulated presentation format (dot arrays, Arabic digits or number words) and reference magnitude (4, 8 or 12). We investigated the effect of presentation format on behavioral results and on brain activation; reference magnitude was manipulated merely to insure sufficient variation in the numerosities used in the task and was not included in analyses.

**Figure 1:**
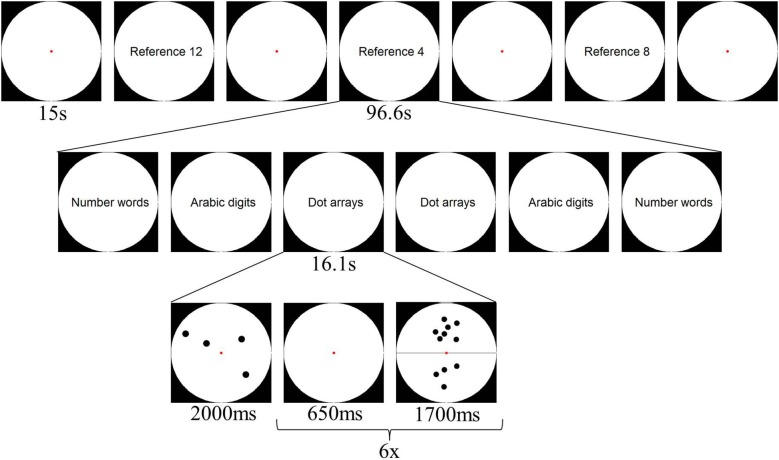
**Schematic overview of a possible design of one experimental run of Experiment 1**.

One experimental run consisted of four fixation blocks of 15 s, alternated with three long reference blocks in which the participant had to compare the result of a subtraction to a specific reference magnitude (4, 8 or 12). Each reference block consisted of six format blocks (two blocks per presentation format) of 16.1 s each. Each of these format blocks consisted of a presentation of the specific reference magnitude in the specific format, and six items in that format. Half of the subtraction items were smaller than the given reference, half of them larger. In total, a run lasted 349.8 s, and participants performed 6 runs. The design is further illustrated in Figure 1.

**Figure 2:**
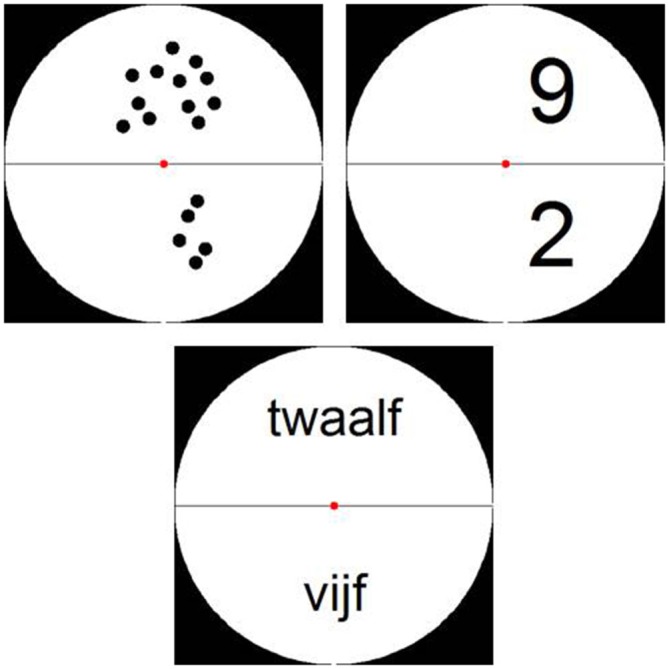
**Examples of stimuli from Experiment 1: stimuli presented as dot arrays, Arabic digits and (Dutch) number words**.

Stimuli were presented in a white circle on a black background divided into two halves by a horizontal black line. The numerosity presented in the lower half of the circle had to be subtracted from the numerosity in the upper half (see Figure 2). Items presented as dot arrays were created via a Matlab script (Dehaene et al., 2005) and were controlled for parameters such as item size, total area and luminance by manipulating dot size. Furthermore, items presented as Arabic digits and number words were controlled for amount of visual information, by varying the font size and position within the circle (see Figure 2). As all participants were Dutch speaking, number words were presented in Dutch. These stimuli were created using an adapted version of the Matlab script by Dehaene et al. (2005).

#### Experiment 2

**Figure 3:**
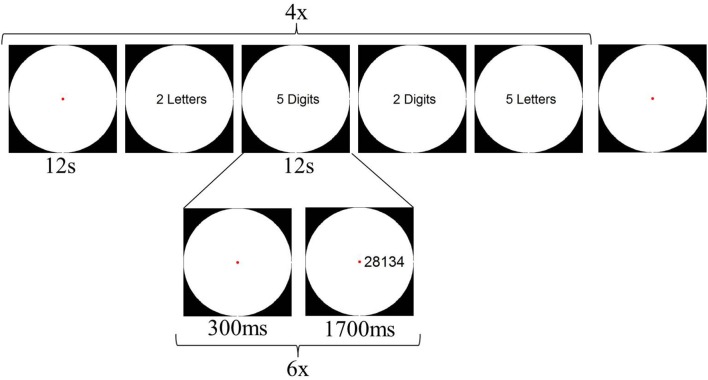
**Schematic overview of a possible design of Experiment 2**.

Although we controlled the stimuli from Experiment 1 for the amount of visual information (i.e., number of black pixels in the stimulus), it is evident that number words always consist of more visual elements (i.e., multiple letters) than digits (i.e., one or two elements). Previous research has shown that visual regions, such as the LOC, can be sensitive to the number of visual elements presented (Xu and Chun, 2006; Xu, 2008). To control for this potential confound of the number of visual elements on the screen, participants performed a second fMRI experiment immediately after Experiment 1. In Experiment 2, both string length (2- or 5-characters) and character format (Arabic digits or letters) were manipulated. Participants performed an order judgment task: They were asked to indicate whether the ordering of the first character relative to the last character was correct or not. In the two digit conditions (both 2- and 5-characters), the ordering was correct if the first character was numerically smaller than the last character. In the letter conditions, alphabetical order was correct. Four blocks per condition alternated with five fixation blocks were presented during this experiment, with each block lasting 12 s. Within each trial block, six stimuli were presented. Total duration of one run was 252 s, and participants performed 4 runs. The design of Experiment 2 is illustrated in Figure 3.

**Figure 4:**
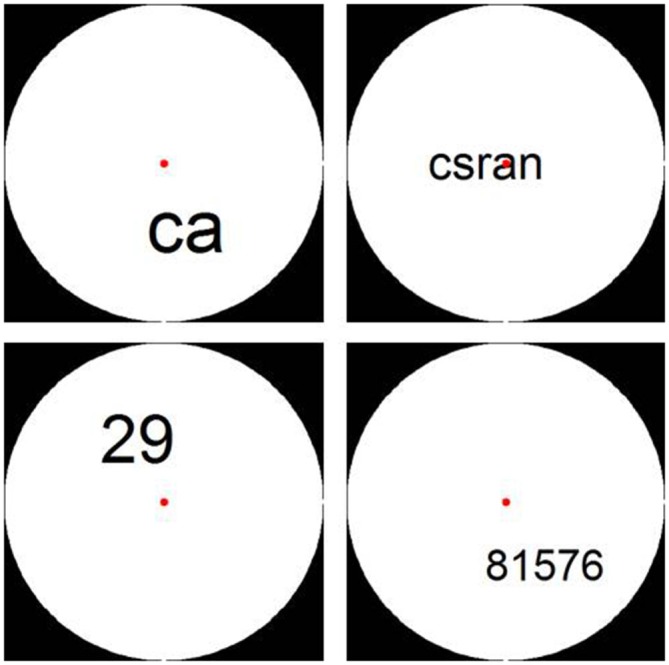
**Examples of stimuli from Experiment 2: 2- and 5-character letter and digit strings**.

Stimuli were presented in a white circle on a black background. For digit conditions, Arabic digits 1–9 were used in the creation of the random digit strings. In the letter conditions, 9 letters, which were visually similar to the digits, were selected: a, c, e, n, r, s, v, x, and z. None of the letter strings represented existing words. The stimuli were again created by adapting the Matlab script by Dehaene et al. (2005). We controlled for visual parameters by varying font size and placement within the circle (see Figure 4).

### Analyses

#### Behavioral Analyses

The behavioral data from both experiments were analyzed with SPSS (IBM SPSS Statistics 22; IBM Corp., Chicago, IL, USA). We controlled for multiple comparisons via a Bonferroni correction. This was done by multiplying each specific *p-*value by the number of contrasts calculated in that analysis. The alpha-criterion therefore remained 0.05.

#### fMRI Preprocessing

All imaging data were preprocessed using the Statistical Parametric Mapping software package (SPM8, Wellcome Department of Cognitive Neurology, London). Functional images were corrected for slice-timing differences, as well as head motion artifacts by realigning all images to the first image. All functional images were coregistered to the anatomical image. Both functional and anatomical images were normalized to the standard Montreal Neurological 152-brain average template. Finally all functional images were spatially smoothed using a Gaussian kernel of 4 mm full-width at half maximum (FWHM). The effect of experimental conditions per voxel was estimated by creating a general linear model per participant. The fixation condition was not explicitly modeled. Motion realignment parameters were included as regressors to control for variation due to movement artifacts.

#### Regions of Interest

To identify different stages of visual processing in each participant, we ran a separate localizer task to localize three visual processing regions of interest: V1, LOC and VWFA. During the localizer runs, words, line drawings of objects, and scrambled lines were presented for 300 ms in a block design. Participants were asked to respond to a word or object if it represented a living entity, and to a scrambled pattern if it was oriented vertically. V1 was localized by selecting all voxels in Brodmann area 17 (located with the anatomical WFU PickAtlas Toolbox, Wake Forrest University PickAtlas, http://fmri.wfubmc.edu/cms/software) that were significantly active in all stimulus conditions vs. fixation. LOC was defined by the contrast [objects—scrambled lines]. Finally, we delineated VWFA by the contrast [words–objects]. The statistical threshold for the ROI selection was *p* < 0.001, uncorrected for multiple comparisons.

#### fMRI Analyses

Both univariate and multivariate correlational analyses were performed on the beta values of the individual conditions as obtained from the general linear model of both fMRI experiments. All functional data were smoothed before the general linear model was estimated (see section fMRI preprocessing), as spatial smoothing is a standard practice for univariate analyses, and is also beneficial for the effect size in correlational multivariate analyses (Op de Beeck, 2010; Brants et al., 2011).

**Figure 5:**
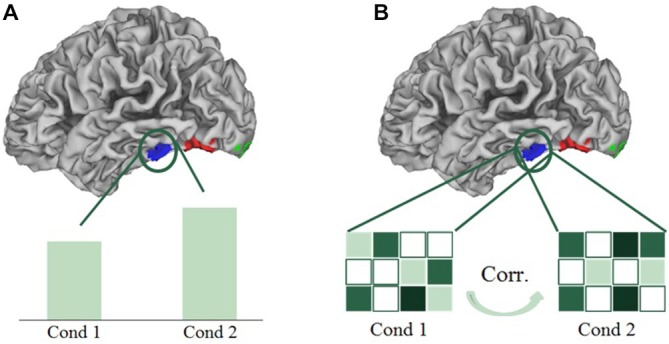
**Schematic presentation of a univariate (A) and multivariate correlational analysis (B)**. In univariate analyses, we averaged the brain activation per condition over all the voxels in a certain region of interest, and compared these mean activations over conditions. In multi-voxel correlational analyses, we correlated the patterns of activation of all conditions.

In univariate analyses, we averaged the brain activation per condition over all the voxels in a certain region of interest, and compared these mean activations (beta values) over conditions (see Figure 5A). In the multi-voxel correlational analyses, we divided the dataset into two halves, and correlated the patterns of activation of all conditions of the first half of the data with the second half, in the delineated regions of interest. This cycle of dividing data and correlating patterns was repeated 100 times; the correlations reported below are the average correlations over those repetitions, which were then transformed via a Fisher-*z* transformation, and were finally averaged over all subjects. In the multi-voxel patterns, the activation of each voxel for each condition was expressed in terms of the beta value of that condition subtracted by the mean beta value across all experimental conditions (“cocktail blank normalization”). Because of the normalization, positive correlations between their activity patterns in a certain brain region indicate more similarity between the corresponding conditions (see Figure 5B). The main advantage of multivariate analyses is that they can reveal differences between conditions that are possibly averaged out in univariate analyses (Norman et al., 2006). To visualize the results obtained from the multivariate correlational analyses, we performed multidimensional scaling (MDS) on the obtained averaged correlation matrices. MDS visualizes the similarity of conditions in 2D-space, with conditions that are represented similarly, and hence have higher correlated activation patterns, presented closer together. Conditions that are represented more distinctly (lower correlated activation patterns) will be shown further apart in the MDS visualization. We also determined the coordinates of the conditions in the MDS plots for each individual subject. These coordinates were then rotated using a Procrustes analysis, to fit the space of the MDS plots of the average correlation matrix. The rotated coordinates of each subject are used as error bars in the MDS plots.

## Results

### Experiment 1

#### Behavioral Results

Mean accuracy and reaction time were calculated over runs and participants (see Table 1). A one-way repeated measures ANOVA with format (dots vs. digits vs. words) as within-subject factor was performed on both the accuracies and the reaction times. Regarding the accuracy scores, we found a main effect of format (*F*_(2,22)_ = 70.79, *p* < 0.001). Pairwise contrasts showed that the accuracy for dot arrays was lower than that for Arabic digits and number words (both *p*’s < 0.001). Accuracy for Arabic digits was significantly higher than that of number words (*p* = 0.01). Turning to the response latencies, we again found a significant main effect of format (*F*_(2,22)_ = 12.65, *p* < 0.001). Participants were significantly faster in responding to dot arrays and Arabic digits than to number words (*p* = 0.03 and *p* < 0.001, respectively). The difference in reaction time between dot arrays and Arabic digits was not significant (*p* = 0.84).

**Table 1:** **Behavioral results Experiment 1**.

	RT (ms)	*SD*	% Correct	*SD*
Dot arrays	1151	146	69.56	9.55
Arabic digits	1108	191	87.58	9.94
Number words	1260	183	82.41	13.29

#### Imaging Results

**Figure 6:**
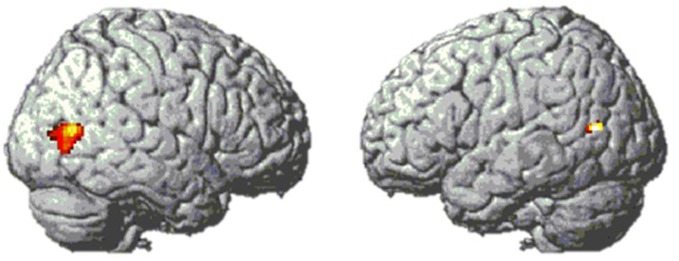
**Bilateral lateral occipital activation clusters from contrast [digits–words] in Experiment 1**. MNI coordinates of peak voxels are [49 −71 9] and [−44 −67 14].

First, we used a whole-brain analysis to determine which regions might be hosting the visual code. Analogous to previous studies (e.g., Pinel and Dehaene, 2013), this was done by comparing the brain activity of Arabic digits vs. words. Both conditions included a symbolic and semantic format with which participants had to calculate, and they mainly differed in the degree to which they were supposed to activate the visual code in the Triple Code Model. There was only one brain region showing higher activity for Arabic numbers than for digits, namely the bilateral lateral occipital cortex (see Figure 6).

The opposite contrast (number words > Arabic digits) showed activation in the occipital lobe around V1, which is probably related to the fact that the number words comprise more characters, and thus extend more to the left and the right with respect to the fixation point than digits.

**Figure 7:**
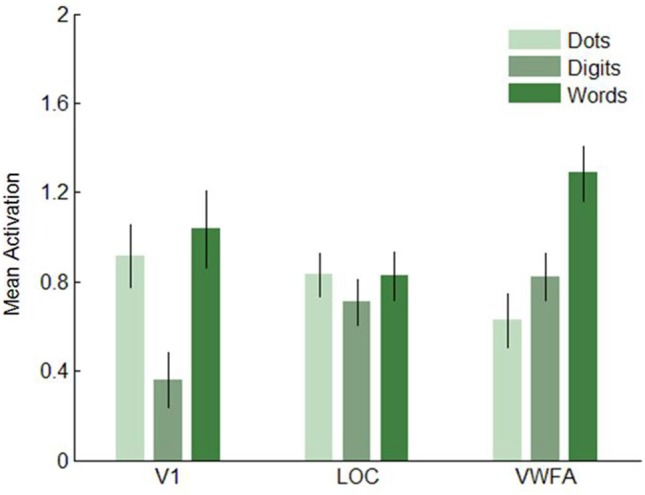
**Mean activation elicited by the three format conditions in the three regions of interest in Experiment 1**. Error bars represent standard error of the mean.

**Figure 8:**
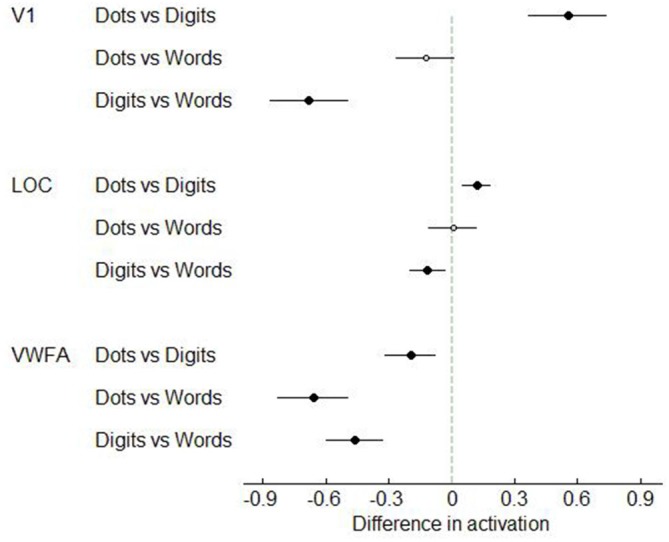
**T-values of the contrasts between conditions in Experiment 1**. Error bars represent a 95% confidence interval. Significant *t*-tests are represented by black, larger dots. *T*-tests that did not reach significance, are represented as open, smaller dots.

Next, we analyzed the data in our three visual regions of interest, i.e., V1, LOC and VWFA. (Figures 7–8). In V1, dot arrays and number words elicited more activation than Arabic digits did (both *p*’s < 0.001), whereas dot arrays and number words did not differ in terms of activation (*p* = 0.07). The same pattern was found, though with smaller effect size, in LOC: dot arrays and number words activated this region more than Arabic digits did (*p* = 0.003 and *p* = 0.01, respectively), whereas dot arrays and number words did not differ in terms of activation (*p* = 0.91). Furthermore, this analysis revealed that the lateral occipital region specifically activated by Arabic symbols was not overlapping with our functionally defined LOC. Finally, in the third* a priori* defined region of interest, the VWFA, we found significantly more activation for number words than for digits (*p* < 0.001), and higher activation for digits than for dot arrays (*p* = 0.005).

### Experiment 2

#### Behavioral Results

Mean reaction time as well as mean accuracy were calculated for each participant and averaged over runs (see Table 2). A two-way repeated measures ANOVA with format (letters vs. digits) and string length (2 vs. 5) as within-subject factors was performed both on the reaction time and the accuracy scores. We found a significant main effect of format (*F*_(1,11)_ = 159.32, *p* < 0.001) and of string length (*F*_(1,11)_ = 88.42, *p* < 0.001) for the reaction time data. Digit strings were solved faster than letter strings, and 2-character strings were solved faster than 5-character strings. Also, the interaction effect between format and string length was significant (*F*_(1,11)_ = 26.78, *p* < 0.001). This effect was driven by a smaller difference between the two letter conditions compared to the two digit conditions. Regarding the accuracy scores, only a main effect of format was found (*F*_(1,11)_ = 26.10, *p* < 0.001), indicating that digit strings were solved more accurately than letter strings. The effect of string length and the interaction effect were not significant (*F*_(1,11)_ = 0.87, *p* = 0.37 and *F*_(1,11)_ = 1.13, *p* = 0.31, respectively).

**Table 2:** **Behavioral results Experiment 2**.

	RT (ms)	*SD*	% Correct	*SD*
2 Letters	956	147	90.02	7.72
5 Letters	996	151	90.03	8.72
2 Digits	792	148	98.26	3.31
5 Digits	885	162	97.05	3.93

#### Imaging Results

**Figure 9:**
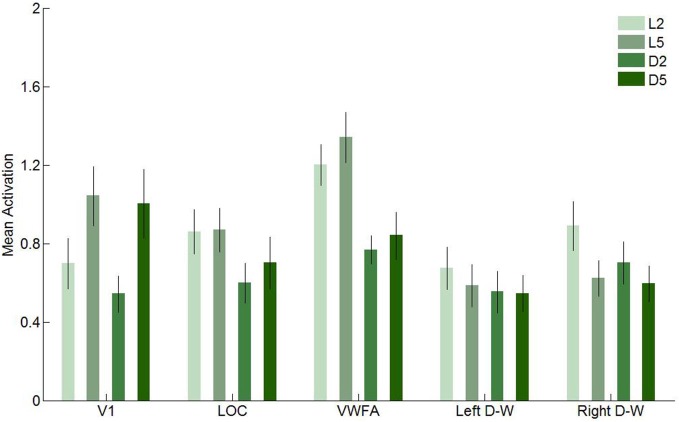
**Mean activation elicited by the four conditions in the five regions of interest in Experiment 2**. Error bars represent standard error of the mean.

In this second experiment, we investigated brain activity in response to our four conditions in five regions of interest: V1, LOC, VWFA and the two [digits–words] clusters found in Experiment 1. These results are presented in Figure 9 (univariate) and in Table 3 and Figure 11 (multivariate).

**Table 3:** **Averaged correlational matrices over all subjects, per region of interest**.

	V1				LOC				VWFA			
	2L	5L	2D	5D	2L	5L	2D	5D	2L	5L	2D	5D
2L	0.41	−0.40	0.36	−0.38	0.15	0.01	0	−0.19	0.20	0.34	−0.26	−0.34
5L	−0.43	0.57	−0.55	0.39	0.02	0.31	−0.31	0	0.34	0.68	−0.60	−0.37
2D	0.37	−0.54	0.58	−0.37	0.01	−0.30	0.29	−0.01	−0.25	−0.63	0.54	0.35
5D	−0.35	0.38	−0.37	0.39	−0.18	0	−0.02	0.20	−0.34	−0.38	0.33	0.37
	**Left [digits–words]**				**Right [digits–words]**			
	**2L**	**5L**	**2D**	**5D**	**2L**	**5L**	**2D**	**5D**
2L	0.43	−0.11	0.02	−0.34	0.23	−0.09	0.08	−0.21
5L	−0.09	0.34	−0.16	0.02	−0.09	0.31	−0.24	0.07
2D	0.01	−0.17	0.13	0.01	0.07	−0.24	0.16	−0.06
5D	−0.35	0.02	0	0.33	−0.22	0.09	−0.07	0.19

We first performed a two-way ANOVA with format (letters and digits) and string length (2- and 5-characters) as within-subject factors in every region of interest. In V1, we found a significant main effect for string length, with higher activity levels for 5-character than for 2-character strings (*F*_(1,11)_ = 23.72, *p* < 0.001), as well as a significant main effect of format (*F*_(1,11)_ = 5.50, *p* = 0.04), indicating that letters elicited more activation in V1 than digits. In LOC, only the effect of format was significant (*F*_(1,11)_ = 64.72, *p* < 0.001). Again, letters elicited more activation than digits. In VWFA, we found a significant main effect of format (*F*_(1,11)_ = 126.70, *p* < 0.001) and of string length (*F*_(1,11)_ = 5.44, *p* = 0.04), with letters and 5-character strings eliciting more activation, respectively. In the left [digits–words] region, the effect of format was significant (*F*_(1,11)_ = 6.38, *p* = 0.03), with letters eliciting higher activation levels, and in the right [digits–words] region, the effect of format, the effect of string length and the interaction effect were significant (*F*_(1,11)_ = 9.46, *p* = 0.01; *F*_(1,11)_ = 9.29, *p* = 0.01; *F*_(1,11)_ = 5.52, *p* = 0.04, respectively).

**Figure 10:**
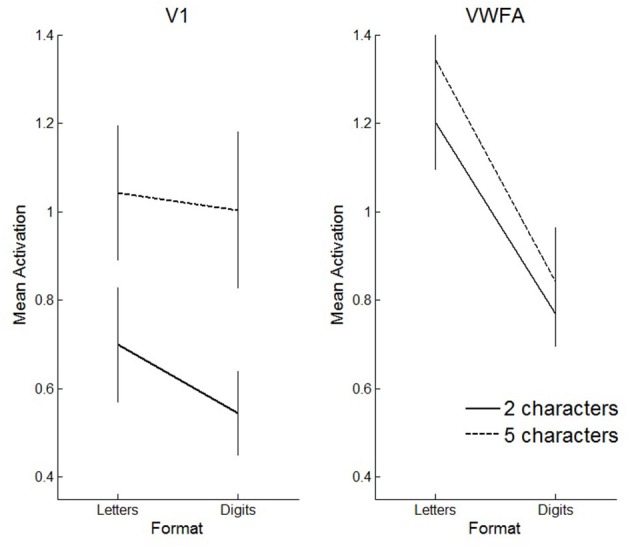
**Visualization of the three-way interaction effect**. Solid lines represent 2-character strings, dashed lines 5-character strings. In primary visual cortex (V1), the effect of string length is clearly more pronounced than the effect of format, whereas in VWFA, the opposite pattern is visible. Error bars represent the standard error of the mean.

We also performed a three-way ANOVA with format (letters vs. digits), string length (2- vs. 5-characters) and region of interest (V1 vs. VWFA) as within subject factors. We found a significant three-way interaction (*F*_(1,11)_ = 7.03, *p* = 0.02). This interaction reflects that in VWFA, the activation increase for letters vs. digits was similar for 5- and 2-character sequences, while in V1 the activation increase for letters vs. digits was stronger for 2-character strings compared to 5-character strings (see Figure 10).

We did not find a significant main effect of format in favor of digits in the [digits–words] regions found in Experiment 1. Thus, the preference for digits over words from Experiment 1 was not replicated in Experiment 2. This indicates that the area observed in Experiment 1 is not specifically sensitive to Arabic digits. In other words, the current data did not confirm the existence of a visual number form area.

Turning to multi-voxel analyses, we found a transformation in the representation of the four conditions across the regions of interest. In all regions of interest, we compared correlations between conditions that only differed in one factor (i.e., same format strings differing in length to look into the effect of string length) with correlations between the condition and itself (located on the diagonal of the correlational matrix, see Table 3). In V1, we found a significant effect of string length (*t*_(11)_ = −4.83, *p* = 0.001), which indicates that activation patterns in V1 are sensitive to the number of characters on the screen. The effect of format was not significant (*t*_(11)_ = −0.91, *p* = 0.38), indicating that V1 is not sensitive to format category. In LOC, both the effect of string length (*t*_(11)_ = −5.42, *p* < 0.001) and the effect of format (*t*_(11)_ = −4.00, *p* < 0.001) were significant. VWFA is sensitive to format (*t*_(11)_ = −5.42, *p* < 0.001), but not to string length (*t*_(11)_ = −1.94, *p* = 0.08). In the left [digits–words] region, we found a significant main effect of string length (*t*_(11)_ = −4.69, *p* < 0.001) and of format (*t*_(11)_ = −2.30, *p* = 0.04). Finally, in the right [digits–words] region, only the effect of string length was significant (*t*_(11)_ = −6.29, *p* < 0.001).

**Figure 11:**
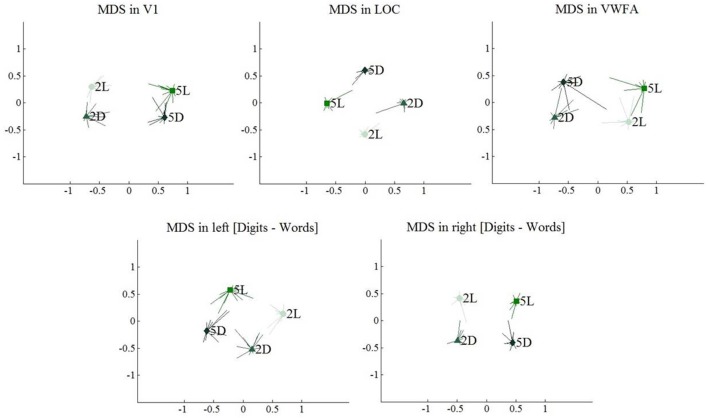
**Multi-dimensional scaling plots, visualizing similarity between multi-voxel activation patterns for the four included conditions**. Light colors represent 2-character strings, dark colors represent 5-character strings. Error bars represent the deviation to the mean per individual subject.

As summarized in the MDS plots in Figure 11, we can conclude that V1 clustered conditions based on the number of characters. In LOC, there was no clear clustering of conditions, there was a sensitivity for both format and string length. In VWFA, we found a clustering based on stimulus category (format). Finally, in the right and left [digits–words] regions we found results similar to those in V1 and LOC, respectively. All these results are visualized on the MDS plots in Figure 11.

## Discussion

The current study served two aims. First, we investigated whether there is a region specifically tuned for Arabic digits that might potentially host the visual code of number processing described in the Triple Code Model (Dehaene and Cohen, 1995, 1997) by contrasting the brain activity elicited by Arabic digits with activity elicited by number words in an arithmetic task. Second, we studied the emergence and formation of this visual code by looking at the evolution of activation patterns throughout the early visual ventral processing stream, more specifically in regions V1, LOC and VWFA.

Two fMRI experiments were conducted. In Experiment 1, participants performed an arithmetic task with subtractions presented in three different formats: Arabic digits, number words and dot arrays. Earlier studies that used subtraction paradigms to investigate the neural correlates of arithmetic have found activation in occipito-temporal areas (for a meta-analysis, see Arsalidou and Taylor, 2011). Similarly, we found a bilateral cluster in lateral occipital cortex that was significantly more active for digits than for number words, which could possibly reflect a region more specifically tuned for digits.

However, the data of Experiment 1 should be treated with great caution. Although we controlled our stimuli for amount of visual information (black pixels) presented, the number of visual elements on the screen varied greatly between digits and number words. This is impossible to control in an arithmetic experiment because number words by definition consist of more visual elements than digits. To ensure that the effects found in Experiment 1 were not due to this difference in visual information, a second fMRI experiment was conducted, in which the number of visual elements presented was controlled for. In Experiment 2, participants were asked to judge the ordering of letter or digit strings, both consisting of either 2- or 5-characters. We reasoned that, if the bilateral [digits–words] region found in Experiment 1 represents a focal region specifically tuned for digits, it should be activated more strongly for digit strings than for letter strings, regardless of string length. However, this was not the case: in both [digits–words] regions, letter strings elicited more activation than digit strings did. Therefore, the data from Experiment 2 revealed that the region observed in Experiment 1 did not show any preference for digits, at least not in a task-independent and string length-independent manner. This leads us to conclude that the findings of Experiment 1 were either due to task-specific effects, or to visual confounds, and that we did *not* find a region hosting the visual code for digits. At most, one could argue that the activation of these [digits–words] regions would be very much task dependent.

Another limitation which is particularly prominent in Experiment 1 is caused by the differences in behavioral performance between conditions. Because we used block designs in both experiments, it was impossible to discard the incorrectly solved trials in the fMRI analyses. It is well established that erroneous responses elicit additional brain activation in regions associated with performance monitoring, such as the anterior cingulate cortex (Carter et al., 1998; Garavan et al., 2002). The effect of this increase in brain activity on visual processes in the context of the present study remains unclear. Future studies should therefore attempt to equalize performance levels over all conditions or should employ an event-related paradigm in which it is possible to only analyze brain activity during the correctly solved items.

The current findings might not be in line with the conclusions from previous studies, but could at least suggest a few variables which should be taken into account in future studies. Park et al. (2012) only found a region activated more by numbers than by letters in the right hemisphere using a same/different-task. Pinel and Dehaene (2013) found a region that was more activated for digits than for number words in the context of a number comparison task, located in the right fusiform gyrus, and Pinel et al. (2001) found a region in inferior temporal gyrus that was part of the arithmetic network in the context of a subtraction task. Importantly, none of these studies controlled neither for variability in the number of visual elements presented nor for task-related factors driving the effect, as we did in the present study. It therefore remains uncertain if the visual regions described in these previous studies are specifically involved in the processing of the visual code.

A study by Shum et al. (2013) suggests that there might be a focal region with a task-independent preference for digits over number words, but that typical fMRI studies, such as the current one, do not have the sensitivity to detect this region. Shum et al. (2013) used intracranial electrophysiological recordings, and found a region in the inferior temporal gyrus that responded more to digits compared to morphologically, phonologically and semantically similar symbols. This possible *visual number form area* was located in a 3T MRI signal drop-out zone, which might explain why we were not able to pick it up using fMRI in healthy adults. Indeed, we inspected our fMRI images and had signal drop-out at the coordinates reported by Shum et al. (2013). However, a recent study by Abboud et al. (2015) reported a number form area in congenitally blind and sighted adults that was located in the right inferior temporal gyrus [53, −44, −12], near the region reported by Shum et al. (2013), but outside of this signal drop-out zone. This region is located far more anteriorly than the region we found in Experiment 1. However, the subject sample, the complex stimuli, and the categorization task Abboud et al. used are very different from our sample, stimuli and subtraction task, making it difficult to compare both studies.

It has been debated in the fMRI literature to what extent it is fruitful to focus exclusively upon small focal regions with a clear preference for a particular stimulus condition, and ignore the large parts of cortex, which are also activated by this condition without a clear preference for other conditions (see e.g., Haxby et al., 2001; Spiridon and Kanwisher, 2002). A second aim of this study was therefore to investigate the emergence of the visual code along the ventral visual processing stream in regions, which might differentially process the different symbolic numerical formats (i.e., digits and number words). We did this by comparing patterns of activation of the four conditions of Experiment 2 in five regions of interest along this visual processing pathway: V1, LOC, VWFA, and the two [digits–words] clusters found in Experiment 1.

In V1, digits and letters of the same string length were clustered, suggesting that V1 clusters stimuli based on amount of visual information. In LOC and in both [digits–words] clusters, we found a less clear picture: all conditions appeared to be represented distinctly with no clear clustering. Nevertheless, each of the ROIs was sensitive to the different conditions. Most strikingly, the left [digits–words] cluster, which was activated similarly by all four conditions according to univariate analyses, still differentiated digit strings from letter strings (see Table 3). Thus, despite the absence of a focal region preferring digit strings over letter strings, there is clear evidence for a distributed selectivity for the difference between digits and letters. This selectivity is most striking in the VWFA, where it is accompanied by a focal preference of the whole region for letters over digits (Polk et al., 2002; Cohen and Dehaene, 2004; Reinke et al., 2008). The representations in VWFA make a categorical distinction between digits and letters and mostly ignore the large physical difference between a 2-character and a 5-character string.

## Conclusion

Based on the results of both Experiment 1 and Experiment 2, we suggest that there is an alteration in representations throughout the ventral processing stream from clustering based on amount of visual information towards clustering based on symbolic stimulus category, as found previously for objects in general (Op de Beeck et al., 2008). The emerging selectivity for the two symbolic formats, digit vs. letter strings, is focal to a certain extent, with task-independent preference for letters over digits in the VWFA and possibly a task-dependent preference for digits over number words in lateral occipital cortex. The emerging selectivity is also distributed across regions, which do not have an overall preference for one format over the other.

## Conflict of Interest Statement

The authors declare that the research was conducted in the absence of any commercial or financial relationships that could be construed as a potential conflict of interest.

## References

[B1] Abboud S. Maidenbaum S. Dehaene S. Amedi A. (2015). A number-form area in the blind. Nat. Commun. 6:6026. 10.1038/ncomms7026 25613599 PMC4338545

[B2] Ansari D. (2008). Effects of development and enculturation on number representation in the brain. Nat. Rev. Neurosci. 9, 278–291. 10.1038/nrn2334 18334999

[B3] Arsalidou M. Taylor M. J. (2011). Is 2+2=4? Meta-analyses of brain areas needed for numbers and calculations. Neuroimage 54, 2382–2393. 10.1016/j.neuroimage.2010.10.009 20946958

[B4] Baker C. I. Liu J. Wald L. L. Kwong K. K. Benner T. Kanwisher N. (2007). Visual word processing and experiential origins of functional selectivity in human extrastriate cortex. Proc. Natl. Acad. Sci. U S A 104, 9087–9092. 10.1073/pnas.0703300104 17502592 PMC1885632

[B5] Brainard D. H. (1997). The psychophysics toolbox. Spat. Vis. 10, 433–436. 10.1163/156856897x00357 9176952

[B6] Brants M. Baeck A. Wagemans J. Op de Beeck H. P. (2011). Multiple scales of organization for object selectivity in ventral visual cortex. Neuroimage 56, 1372–1381. 10.1016/j.neuroimage.2011.02.079 21376816

[B7] Bulthé J. De Smedt B. Op de Beeck H. P. (2014). Format-dependent representations of symbolic and non-symbolic numbers in the human cortex as revealed by multi-voxel pattern analyses. Neuroimage 87, 311–322. 10.1016/j.neuroimage.2013.10.049 24201011

[B8] Carter C. S. Braver T. S. Barch D. M. Botvinick M. M. Noll D. Cohen J. D. (1998). Anterior cingulate cortex, error detection and the online monitoring of performance. Science 280, 747–749. 10.1126/science.280.5364.747 9563953

[B9] Cohen L. Dehaene S. (2004). Specialization within the ventral stream: the case for the visual word form area. Neuroimage 22, 466–476. 10.1016/j.neuroimage.2003.12.049 15110040

[B10] Dehaene S. Cohen L. (1995). Towards an anatomical and functional model of number processing. Math. Cogn. 1, 83–120.

[B11] Dehaene S. Cohen L. (1997). Cerebral pathways for calculation: double dissociation between rote verbal and quantitative knowledge of arithmetic. Cortex 33, 219–250. 10.1016/s0010-9452(08)70002-9 9220256

[B12] Dehaene S. Izard V. Piazza M. (2005). Control over non-numerical parameters in numerosity experiments. Unpublished manuscript. (Available online at www.unicog.org)

[B13] Dehaene S. Piazza M. Pinel P. Cohen L. (2003). Three parietal circuits for number processing. Cogn. Neuropsychol. 20, 487–506. 10.1080/02643290244000239 20957581

[B14] Delazer M. Domahs F. Bartha L. Brenneis C. Lochy A. Trieb T.. (2003). Learning complex arithmetic—an fMRI study. Brain Res. Cogn. Brain Res. 18, 76–88. 10.1016/j.cogbrainres.2003.09.005 14659499

[B15] Eger E. Michel V. Thirion B. Amadon A. Dehaene S. Kleinschmidt A. (2009). Deciphering cortical number coding from human brain activity patterns. Curr. Biol. 19, 1608–1615. 10.1016/j.cub.2009.08.047 19781939

[B16] Garavan H. Ross T. J. Murphy K. Roche R. A. Stein E. A. (2002). Dissociable executive functions in the dynamic control of behavior: inhibition, error detection and correction. Neuroimage 17, 1820–1829. 10.1006/nimg.2002.1326 12498755

[B17] Goodale M. A. Milner A. D. (1992). Separate visual pathways for perception and action. Trends Neurosci. 15, 20–25. 10.1016/0166-2236(92)90344-8 1374953

[B18] Grabner R. H. Ansari D. Koschutnig K. Reishofer G. Ebner F. Neuper C. (2009). To retrieve or to calculate? Left angular gyrus mediates the retrieval of arithmetic facts during problem solving. Neuropsychologia 47, 604–608. 10.1016/j.neuropsychologia.2008.10.013 19007800

[B19] Grill-Spector K. Kourtzi Z. Kanwisher N. (2001). The lateral occipital complex and its role in object recognition. Vision Res. 41, 1409–1422. 10.1016/s0042-6989(01)00073-6 11322983

[B20] Grill-Spector K. Malach R. (2004). The human visual cortex. Annu. Rev. Neurosci. 27, 649–677. 10.1146/annurev.neuro.27.070203.144220 15217346

[B21] Halgren E. Dale A. M. Sereno M. I. Tootell R. B. Marinkovic K. Rosen B. R. (1999). Location of human face-selective cortex with respect to retinotopic areas. Hum. Brain Mapp. 7, 29–37. 10.1002/(sici)1097-0193(1999)7:1<29::aid-hbm3>3.0.co;2-r 9882088 PMC6873292

[B22] Haxby J. V. Gobbini M. I. Furey M. L. Ishai A. Schouten J. L. Pietrini P. (2001). Distributed and overlapping representations of faces and objects in ventral temporal cortex. Science 293, 2425–2430. 10.1126/science.1063736 11577229

[B23] Keller K. Menon V. (2009). Gender differences in the functional and structural neuroanatomy of mathematical cognition. Neuroimage 47, 342–352. 10.1016/j.neuroimage.2009.04.042 19376239 PMC2888277

[B24] Malach R. Levy I. Hasson U. (2002). The topography of high-order human object areas. Trends Cogn. Sci. 6, 176–184. 10.1016/s1364-6613(02)01870-3 11912041

[B25] Menon V. (2015). “Arithmetic in the child and adult brain,” in The Oxford Handbook of Numerical Cognition, eds Cohen Kadosh R. Dowker A. (Oxford, UK: Oxford University Press), 502–530.

[B26] Mishkin M. Ungerleider L. G. Kathleen A. (1983). Object vision and spatial vision: two cortical pathways. Trends Neurosci. 6, 414–417. 10.1016/0166-2236(83)90190-x

[B27] Nieder A. Dehaene S. (2009). Representation of number in the brain. Annu. Rev. Neurosci. 32, 185–208. 10.1146/annurev.neuro.051508.135550 19400715

[B28] Norman K. A. Polyn S. M. Detre G. J. Haxby J. V. (2006). Beyond mind-reading: multi-voxel pattern analysis of fMRI data. Trends Cogn. Sci. 10, 424–430. 10.1016/j.tics.2006.07.005 16899397

[B29] Op de Beeck H. P. (2010). Against hyperacuity in brain reading: spatial smoothing does not hurt multivariate fMRI analyses? Neuroimage 49, 1943–1948. 10.1016/j.neuroimage.2009.02.047 19285144

[B30] Op de Beeck H. P. Haushofer J. Kanwisher N. G. (2008). Interpreting fMRI data: maps, modules and dimensions. Nat. Rev. Neurosci. 9, 123–135. 10.1038/nrn2314 18200027 PMC2731480

[B31] Park J. Hebrank A. Polk T. A. Park D. C. (2012). Neural dissociation of number from letter recognition and its relationship to parietal numerical processing. J. Cogn. Neurosci. 24, 39–50. 10.1162/jocn_a_00085 21736455 PMC3357212

[B32] Piazza M. Pinel P. Le Bihan D. Dehaene S. (2007). A magnitude code common to numerosities and number symbols in human intraparietal cortex. Neuron 53, 293–305. 10.1016/j.neuron.2006.11.022 17224409

[B33] Pinel P. Dehaene S. (2013). Genetic and environmental contributions to brain activation during calculation. Neuroimage 81, 306–316. 10.1016/j.neuroimage.2013.04.118 23664947

[B34] Pinel P. Dehaene S. Rivière D. LeBihan D. (2001). Modulation of parietal activation by semantic distance in a number comparison task. Neuroimage 14, 1013–1026. 10.1006/nimg.2001.0913 11697933

[B36] Polk T. A. Stallcup M. Aguirre G. K. Alsop D. C. D’Esposito M. Detre J. A.. (2002). Neural specialization for letter recognition. J. Cogn. Neurosci. 14, 145–159. 10.1162/089892902317236803 11970782

[B37] Price G. R. Ansari D. (2011). Symbol processing in the left angular gyrus: evidence from passive perception of digits. Neuroimage 57, 1205–1211. 10.1016/j.neuroimage.2011.05.035 21620978

[B38] Raizada R. D. S. Kriegeskorte N. (2010). Pattern-information fMRI: new questions which it opens up and challenges which face it. Int. J. Imaging Syst. Technol. 20, 31–41. 10.1002/ima.20225

[B39] Reddy L. Kanwisher N. (2006). Coding of visual objects in the ventral stream. Curr. Opin. Neurobiol. 16, 408–414. 10.1016/j.conb.2006.06.004 16828279

[B40] Reinke K. Fernandes M. Schwindt G. O’Craven K. Grady C. L. (2008). Functional specificity of the visual word form area: general activation for words and symbols but specific network activation for words. Brain Lang. 104, 180–189. 10.1016/j.bandl.2007.04.006 17531309

[B41] Rickard T. C. Romero S. G. Basso G. Wharton C. Flitman S. Grafman J. (2000). The calculating brain: an fMRI study. Neuropsychologia 38, 325–335. 10.1016/s0028-3932(99)00068-8 10678698

[B42] Rosenberg-Lee M. Chang T. T. Young C. B. Wu S. Menon V. (2011). Functional dissociations between four basic arithmetic operations in the human posterior parietal cortex: a cytoarchitectonic mapping study. Neuropsychologia 49, 2592–2608. 10.1016/j.neuropsychologia.2011.04.035 21616086 PMC3165023

[B43] Rossion B. Caldara R. Seghier M. Schuller A. M. Lazeyras F. Mayer E. (2003). A network of occipito-temporal face-sensitive areas besides the right middle fusiform gyrus is necessary for normal face processing. Brain 126, 2381–2395. 10.1093/brain/awg241 12876150

[B44] Shum J. Hermes D. Foster B. L. Dastjerdi M. Rangarajan V. Winawer J.. (2013). A brain area for visual numerals. J. Neurosci. 33, 6709–6715. 10.1523/jneurosci.4558-12.2013 23595729 PMC3970733

[B45] Simon O. Mangin J. F. Cohen L. Le Bihan D. Dehaene S. (2002). Topographical layout of hand, eye, calculation and language-related areas in the human parietal lobe. Neuron 33, 475–487. 10.1016/s0896-6273(02)00575-5 11832233

[B46] Spiridon M. Kanwisher N. (2002). How distributed is visual category information in human occipito-temporal cortex? An fMRI study. Neuron 35, 1157–1165. 10.1016/s0896-6273(02)00877-2 12354404

[B47] Wu S. S. Chang T. T. Majid A. Caspers S. Eickhoff S. B. Menon V. (2009). Functional heterogeneity of inferior parietal cortex during mathematical cognition assessed with cytoarchitectonic probability maps. Cereb. Cortex 19, 2930–2945. 10.1093/cercor/bhp063 19406903 PMC2774395

[B48] Xu Y. (2008). Representing connected and disconnected shapes in human inferior intraparietal sulcus. Neuroimage 40, 1849–1856. 10.1016/j.neuroimage.2008.02.014 18353688

[B49] Xu Y. Chun M. M. (2006). Dissociable neural mechanisms supporting visual short-term memory for objects. Nature 440, 91–95. 10.1038/nature04262 16382240

[B50] Zago L. Pesenti M. Mellet E. Crivello F. Mazoyer B. Tzourio-Mazoyer N. (2001). Neural correlates of simple and complex mental calculation. Neuroimage 13, 314–327. 10.1006/nimg.2000.0697 11162272

